# Designing a strategy to implement optimal conservative treatments in patients with knee or hip osteoarthritis in orthopedic practice: a study protocol of the BART-OP study

**DOI:** 10.1186/1748-5908-9-22

**Published:** 2014-02-18

**Authors:** Stefanie N Hofstede, Thea PM Vliet Vlieland, Cornelia HM van den Ende, Perla J Marang-van de Mheen, Rob GHH Nelissen, Leti van Bodegom-Vos

**Affiliations:** 1 Department of Medical Decision Making, Leiden University Medical Center, Albinusdreef 2, Leiden, 2333 ZA The Netherlands; 2 Department of Orthopaedics, Leiden University Medical Center, Albinusdreef 2, Leiden, 2333 ZA The Netherlands; 3 Department of Rheumatology, Sint Maartenskliniek Nijmegen, Hengstdal 3, Ubbergen, 6574 NA The Netherlands

**Keywords:** Osteoarthritis, Hip, Knee, Conservative treatment, Implementation strategy, Barriers and facilitators

## Abstract

**Background:**

National and international evidence-based guidelines for hip and knee osteoarthritis recommend to start with (a combination of) conservative treatments, followed by surgical intervention if a patient does not respond sufficiently to conservative treatment options. Despite these recommendations, there are strong indications that conservative treatments are not optimally used in orthopedic practice. Our study aims to quantify the use of conservative treatments in Dutch orthopedic practice and to explore the barriers and facilitators for the use of conservative treatments that should be taken into account in a strategy to improve the embedding of conservative treatments in hip and knee osteoarthritis in orthopedic practice.

**Methods:**

This study consists of three phases. First, current use of conservative treatments in patients with hip and knee osteoarthritis will be explored using an internet-based survey among at least 100 patients to identify the underused conservative treatments. Second, barriers and facilitators for the use of conservative treatments in orthopedic practice will be identified using semi-structured interviews among 10 orthopedic surgeons and 5 patients. The interviews will be followed by an internet-based survey among approximately 450 orthopedic surgeons and at least 100 patients in which the identified barriers and facilitators will be ranked by importance. Finally, an implementation strategy will be developed based on the results of the previous phases using intervention mapping.

**Discussion:**

The developed strategy is likely to result in an optimal and standardized use of conservative treatment options in hip and knee osteoarthritis in orthopedic practice, because it is focused on identified barriers and facilitators. In addition, the results of this study can be used as an example for optimizing the use of conservative care in other patient groups. In a subsequent study, the developed implementation strategy will be assessed on its effectiveness, feasibility and costs.

## Background

Osteoarthritis (OA) is a degenerative joint disease primarily characterized by progressive loss of articular cartilage. It leads to pain and loss of function [1]. Approximately 10% of men and 18% of women older than 60 years have OA [2]. Symptomatic OA of the knee and the hip have the highest prevalence within the group of arthritis. Due to the ageing society and obesity, the prevalence of hip and knee OA is still increasing [3].

In 2009, 154 patients per 100,000 persons received a Total Hip Arthroplasty (THA) or Total Knee Arthroplasty (TKA), and 118 patients per 100,000 persons received a TKA in Western countries [4]. However, the lifespan of a prosthesis is limited. The revision rate after a TKA or THA is 12.9% after 10 years [5], and revision arthroplasty is less successful than primary TKA or THA [6]. Therefore, it is important to delay the primary TKA or THA by optimizing the use of conservative treatment options, especially in young people.

National and international evidence-based guidelines for hip and knee OA recommend to start with (a combination of) conservative treatments [7-11]. Conservative treatments include pharmacological options (*e.g*., the use of analgesics, non-steroidal anti-inflammatory drugs, and steroid injection therapy) and non-pharmacological options (*e.g.*, physical therapy, patient education, and wait loss intervention). Conservative treatments aim to prevent progression and reduce symptoms such as joint pain and impairment of functions [11]. If persons do not respond (sufficiently) to conservative treatment options, joint replacement (*i.e*., THA or TKA) can be considered. Despite the recommendation in guidelines to start with conservative treatments and only use surgical intervention if a patient does not respond sufficiently to conservative treatment options, the use of conservative treatments in daily practice is suboptimal [12-15]. For example, a study showed that conservative treatments were not fully exploited in 81% of the patients who were referred to specialized knee/hip OA outpatient clinics [12]. Information about conservative treatments patients receive in orthopedic practice is lacking. Furthermore, surgery rates are rising [16]. TKA and THA in patients with OA increased with 196% and 50% respectively between 1995 and 2005 in the Netherlands [16]. In addition, large variation exists in preoperative status (*e.g*., disease severity) across different centers in Europe and Australia, which suggests differences in the timing of surgery [17,18]. Optimal use of conservative treatments could reduce these differences.

A few models of care were developed to optimize the use of conservative treatments. In Australia, a clinical pathway model and clinician and patient toolkits were developed to support implementation of nonsurgical management of hip and knee OA [19]. However, in Australia, rheumatologists play a leading role, while in the Netherlands, the orthopedic surgeon is responsible for OA treatment in hospital care. In the Netherlands, a stepped-care strategy (SCS) based on (inter)national guidelines [20,21] is developed to facilitate the use of conservative treatments in three steps in primary care [22,23]. The first step consists of education, life style advice, and acetaminophen. If the treatment options in the first step are not sufficient, treatment options in the second step can be considered (exercise therapy, dietary therapy, and non-steroidal anti-inflammatory drugs). Multidisciplinary care, intra-articular injections, and transcutaneous electrical nerve stimulation are treatment options in the third step and could be considered if treatment options in step one or two are ineffective. After implementation of the SCS, most recommended conservative treatments seem to be well used, except dietary therapy [23]. Both studies provide evidence to promote the use of conservative treatments in primary care or in a setting where the rheumatologists play a leading role, but strategies for the optimization of conservative treatments in orthopedic care are still lacking. Information about the current use of conservative treatments, and barriers and facilitators influencing the adoption of conservative treatments in orthopedic practice, is needed to develop a tailored implementation strategy focused on orthopedic care.

In the Netherlands, patients with OA are usually treated by the general practitioner. According to guidelines, patients should be referred to the orthopedic surgeon if they do not respond sufficiently to conservative treatment options. In orthopedic practice, the decision will be made to start/continue conservative treatments or to perform a surgery depending on previous received treatments and disease severity. The leading role of an orthopedic surgeon could result in other barriers and facilitators compared to a setting where the rheumatologists play a leading role. This subsequently results in another strategy to improve the embedding of conservative therapies in hip and knee OA in orthopedic practice. While rheumatologists and general practitioners only provide conservative treatments in OA, orthopedic surgeons can provide both conservative treatments and surgical interventions. It is unclear to what extent factors such as lack of information about conservative treatment options, increasing number of orthopedic surgeons [16], or patient preferences play a role. It is important to explore these factors for the development of a tailored implementation strategy, so that orthopedic surgeons will provide underused treatment options in primary care, such as dietary therapy. Part of this implementation strategy could be the SCS or a clinical pathway model as used in previous implementations.

### Objective

The BART-OP study (Beating osteoARThritis in the Orthopedic Practice) aims to quantify the use of conservative treatments in Dutch orthopedic practice before THA or TKA and to explore the barriers and facilitators for the use of conservative treatments that should be taken into account in a strategy to improve the embedding of conservative treatments in hip and knee OA in orthopedic practice.

To reach the aim of this study, we formulated the following research questions:

1. What is the current use of conservative treatments, before patients receive a surgery, in orthopedic practice?

2. Which barriers and facilitators influence the use of conservative treatments in orthopedic practice?

3. What is an appropriate tailored implementation strategy for the embedding of conservative treatments in orthopedic practice?

In a subsequent study, the developed implementation strategy will be assessed on its effectiveness, feasibility and costs.

## Methods

This study consists of three phases to be executed in one year:

A. The analysis of current use of conservative treatments, before patients receive a surgery in orthopedic practice (months 1 to 9).

B. Identification of barriers and facilitators for non-optimal conservative treatments, using two steps (months 1 to 9).

i. Barriers and facilitators for non-optimal conservative treatments are explored with interviews among orthopedic surgeons and patients.

i. Identified barriers and facilitators are ranked by importance in a survey among a representative sample of orthopedic surgeons and patients.

C. The development of the implementation strategy based on the results of phases A and B (months 9 to 12).

The study design, study population, analysis and outcome measures are described per study phase.

### Phase A. The analysis of current use of conservative treatments before patients receive a surgery in orthopedic practice

#### Study design

To analyze the current use of conservative treatments, before patients undergo THA or TKA in orthopedic practice, an internet-based survey among patients will be performed. The survey will include questions about which conservative treatment options are used before surgery. This information is needed to be able to focus the implementation strategy on the right conservative treatments. The content of the survey will be developed based on the Dutch guideline of OA of the hip and knee [11]. Reminders to non-responders will be sent after two weeks and again after four weeks.

### Study population

The survey will be sent to a sample of at least 100 patients living in different regions of the Netherlands. Inclusion criteria for patients are: age ≥18 years, a doctor’s diagnosis of hip or knee OA, and who have had a TKA or THA no longer than 12 months ago or are on the waiting list for surgery within the next 3 months. Patients with an inability to understand written Dutch will be excluded from the study. We will sample these patients using advertisements in local newspapers, and at websites or newsletters of patient associations.

### Analysis

Descriptive statistics will be used to describe the current use of conservative treatment options in orthopedic practice. Independent t-tests or Mann Whitney U tests for continuous variables and Chi square tests or Fisher’s exact tests for proportions are used to analyze differences in the frequency of use between different regions or other conditions.

### Outcome measures

The main outcome measure is the percentage of patients in whom the conservative treatment options are applied optimally before they undergo surgery, as described in the guideline. These results will help us to focus the implementation strategy, developed in phase C, on the right conservative treatments.

### Phase B. Identification of barriers and facilitators for non-optimal treatment

#### Study design

Two steps will be taken to identify barriers and facilitators associated with the non-optimal use of conservative treatments. First, semi-structured interviews among orthopedic surgeons and patients will be performed to explore all relevant barriers and facilitators for non-optimal conservative therapy. The interview questions will be based on the Theoretical Domains Interview framework (TDI) [24]. The TDI framework includes 12 theoretical construct domains derived from 33 psychological theories and covering 128 explanatory constructs that enhance implementation of evidence-based practice [24]. In addition, barriers and facilitators reported in a previous study about the use of the SCS to optimize hip and knee OA in primary care [25] are included in the interview questions. Second, an internet-based survey will be held among a selection of orthopedic surgeons (n≈400) and sample of patients (n≥100) to rank barriers and facilitators identified in the interviews on importance. The survey will include questions to determine which of these barriers and facilitators are associated with the use of conservative treatments.

### Study population

For the semi-structured interviews, we anticipate interviewing 10 orthopedic surgeons involved in hip and knee surgery and 5 patients who have had a THA or TKA no longer than 12 months ago (≥18 years, and able to understand oral Dutch). If we do not reach data saturation after these interviews (three consecutive interviews without new barriers or facilitators [26]), we will continue interviewing until data saturation is reached. To obtain contrasting views on barriers and facilitators, we will apply purposive sampling. First, we will purposively select orthopedic surgeons and patients from Dutch regions with high surgery rates and from Dutch regions with relatively low surgery rates based on the report of Van Beek *et al*. about variation in clinical practice [27]. In addition, we will select orthopedic surgeons in such a way as to ensure diversity of hospital type (public hospitals and academic hospitals). It is important to include orthopedic surgeons of public and academic hospitals, because this may reveal other facilitators and barriers. For the internet-based survey, Dutch orthopedic surgeons listed in the registry of the Dutch Orthopedic Association (NOV) or the Dutch medical address book will be approached for participation. Inclusion criteria are: involved in hip or knee OA, and access to email address. Patients (n≥100) are recruited using advertisements in local newspapers. Included are patients: ≥18 years who have had total hip or knee surgery no longer than 12 months ago, or are on the waiting list for receiving a THA or TKA. Patients with an inability to understand oral Dutch will be excluded from the study.

### Analysis

The semi-structured interviews will be audio-taped and transcribed in full for analysis. The interviews will be analyzed by two researchers using open coding to ensure that we find all barriers and facilitators for the non-optimal use of conservative therapy. This qualitative analysis will be executed using the software package ATLAS.ti (ATLAS.ti Scientific Software Development GmBH, Berlin, Germany) for this qualitative analysis.

The subsequent survey data will allow us to rank the importance of barriers and facilitators and their relationship with the use of conservative treatments. These relationships will be assessed using multiple regression analysis. We will use SPSS 20.0 for analysis.

### Outcome measures

A list of the most relevant barriers and facilitators for the optimal use of conservative treatments in orthopedic practice before patients with hip or knee OA receive THA or TKA.

### C. The development of the implementation strategy

#### Study design

The results of the previous phases will be used to develop a tailored implementation strategy for the optimal use of conservative treatments in orthopedic practice in patients with hip or knee OA. The results of phase A will show at which type(s) of conservative treatment the strategy should be aimed. Phase B results will show the most relevant barriers and facilitators that should be taken into account in the development of the strategy. From literature, it is known that, in general, multifaceted strategies are more effective than single strategies [28,29]. Assuming this, and our expectation that several barriers on different theoretical domains will be found, it is very likely that the developed implementation strategy includes several components directed at different levels (*i.e*., knowledge or social influences). Furthermore, it is expected that the strategy components will include educational outreach, an interactive educational strategy, and/or patient-specific strategies, because these facets seem to be promising for implementation [28].

In the development process, the project team will use the intervention mapping approach of Bartholomew *et al*. [30]. This method begins with the creation of matrices, in which the performance objectives are set against the most important factors that hinder or facilitate the adoption of conservative treatments. Subsequently, the project team will brainstorm about the strategy components needed to achieve the performance objective in the presence of the barrier or facilitator mentioned in the matrix. The cells of the matrices are then gradually filled with implementation strategy components [30]. Next, the project team will translate the formulated strategy components into practical strategies.

### Analysis

The study group meeting will be summarized. The project members will receive a summary of the meeting and the formulated implementation strategy and will be asked whether the summary and implementation strategy is consistent with the conclusions reached in the meeting.

### Outcome measures

A tailored implementation strategy for the embedding of conservative treatments in orthopedic practice in patients with hip or knee OA.

### Ethical approval

This study protocol was presented to the Medical Ethical Committee of the Leiden University Medical Center (CME P13.087/NV/nv). An exemption was obtained, as ethical approval for this type of study is not required under Dutch law.

## Discussion

The goal of this study is to develop a tailored implementation strategy to optimize the use of conservative treatments in hip and knee OA in patients referred to the orthopedic surgeon.

Several studies have been performed to develop and test implementation strategies, including identification of barriers that prevent implementation [31-33]. They all conclude that a prior inventory of barriers to develop a tailored implementation strategy is useful and can confirm whether barriers differ in different settings. Prior inventory thereby reduces the number of costly trials evaluating different implementation strategies [28,32,34]. Although previous studies already explored barriers for the use of conservative treatments, these studies were performed in other settings, and not focused on orthopedic care. Furthermore, the uptake of several implementation activities was poor, since only 9% of the participating GPs were present at the seminar [23]. It was very difficult to reach all GPs in seminars. This could be easier in orthopedic practice. Orthopedic surgeons may have more interest in OA, because it is part of their specialization, whereas for GPs it is one of the many health problems in their daily practice. This highlights the importance of optimizing the use of conservative treatments in orthopedic practice as well, so that patients will receive optimal treatment options in orthopedic practice if conservative care was suboptimal in their primary care trajectory. Our study and the study performed in primary care together will provide useful information for the development of interventions based on the full spectrum of barriers and facilitators in primary care and orthopedic practice. This is important because a multidisciplinary approach is likely to be more effective to obtain optimal conservative therapy [35].

A strength of this study is the purposive sampling of orthopedic surgeons of regions with low and high surgery rates, because they could have contrasting views on barriers and facilitators. We think that this will reveal most barriers and facilitators for the implementation of the optimal use of conservative treatments in hip and knee OA in orthopedic practice. A limitation may be the selection of patients. Patients will be recruited via advertisement, which can lead to selection bias, because patients who respond to the advertisements may perceive other barriers and facilitators as most important compared to non-responders. Furthermore, the use of an internet-based survey could also induce selection bias. Knee and hip OA increases with age [3], but not all elderly persons do have internet or an email address. This can lead to the selection of younger persons compared to the average age of OA patients, while elderly persons may perceive other barriers and facilitators as most important. We will assess the impact of selection bias by comparing elderly respondents with younger ones. If they perceive the same barriers and facilitators, we can conclude that the impact of this type of selection bias does not influence our results.

The developed strategy is likely to result in an optimal and standardized use of conservative treatment options in hip and knee OA in orthopedic practice. In addition, the results of this study can be used as an example for optimizing the use of conservative care in other patient groups. In a subsequent study, the developed implementation strategy will be assessed on its effectiveness, feasibility and costs.

## Abbreviations

OA: Osteoarthritis; SCS: Stepped-care strategy; THA: Total hip arthroplasty; TKA: Total knee arthroplasty; ZonMw: The Netherlands organization for Health Research and Development.

## Competing interests

The authors declare that they have no competing interests.

## Authors’ contributions

LB, PM and TV designed the study protocol. SH wrote the manuscript and will carry out the study. LB and TV will supervise the study and writing of the manuscript. All authors have critically read and modified both the study protocol and previous drafts of the manuscript, and have approved the final version.
